# A follow-up cohort study on the risk of prediabetes, comparing women with previous preeclamptic or normotensive pregnancies

**DOI:** 10.1038/s41598-023-43014-z

**Published:** 2023-09-30

**Authors:** Louise B. Möller, Marie K. Bladh, Kerstin Brismar, Klara Palm, Ellika G. Andolf

**Affiliations:** 1 Department of Obstetrics and Gynaecology, Department of Clinical and Experimental Medicine, Linköping University, 581 85 Linköping, Sweden; 2 Department of Molecular Medicine and Surgery, Karolinska University Hospital, Karolinska Institutet, 171 76 Stockholm, Sweden; 3 Division of Obstetrics and Gynaecology, Department of Clinical Sciences, Danderyd University Hospital, Karolinska Institutet, Stockholm, Sweden

**Keywords:** Medical research, Risk factors

## Abstract

Studies have shown that preeclampsia is associated insulin resistance and cardiovascular events later in life. However, knowledge is lacking regarding a possible association between PE and abnormal glucose tolerance/prediabetes. Thus, the current study aimed to compare the prevalence of prediabetes in women with previous severe preeclampsia to women with previous normotensive pregnancies. Women with severe preeclampsia (index women, n = 45) admitted to Danderyds University Hospital in 1999–2004 were compared to women with normotensive pregnancies, matched for age, parity, and year of delivery (control women, n = 53). In 2013–2016 BMI, blood pressure, waist circumference, insulin, C-peptide, hsCRP, Cystatin C, HDL, triglycerides, and HbA1c were measured and an OGTT was performed. Index women had a higher BMI (*p* < 0.001) and blood pressure (*p* < 0.001) in early pregnancy. At follow-up, prediabetes was more common among index women (*p* = 0.001), as were hypertension (*p* = 0.003), heredity for diabetes/cardiovascular disease (*p* = 0.020), and a larger waist circumference (*p* = 0.024). Preeclampsia increased the risk of having a fasting plasma glucose ≥ 5.6 mmol/l (aOR 7.28, 95% CI 2.44–21.76) and of prediabetes 11–16 years after index pregnancy (aOR 4.83, 95% CI 1.80–12.97). In conclusion, preeclampsia increases the risk of prediabetes independent of heredity, hypertension, and waist circumference. These findings may have implications for screening and prevention.

## Introduction

Several epidemiological studies have linked preeclampsia (PE) with later cardiovascular events^1–4^, but less attention has been paid to an association between PE and abnormal glucose tolerance (AGT). PE is associated with insulin resistance^5–9^ and women with PE have an increased risk of developing type 2 diabetes later in life^10–14^. However, to our knowledge, only one study has described the risk of developing prediabetes after hypertensive pregnancies^15^. In that study, a significantly higher prevalence of prediabetes four years after index delivery was reported among women with PE than in women with gestational hypertension and normotensive pregnancies. Further, the odds of prediabetes were higher in women with PE and preterm delivery prior to 37 weeks of gestational age^15^.

It is not entirely known whether the association between PE and type 2 diabetes reflects shared common risk factors and pathogenic pathways, a causal relationship between the two, or both^8,10^. Exactly why and when an AGT develops, in a group of what can be considered young women, remains not fully understood. Altogether, the relationship between PE and subsequent AGT entails further study to clarify the association between the two. This is especially important since the prediabetic period provides a window of opportunity for preventative interventions. Prediabetes is not only a risk for type 2 diabetes but also of cardiovascular disease (CVD)^16^. Increased knowledge concerning the risk of prediabetes after PE could have important implications in screening, risk stratification, and management of women all over the world. In the broader perspective, it could be applicable in reducing the global burden of type 2 diabetes and CVD, which is of extreme importance.

The primary objective of this study was to investigate the prevalence of prediabetes 11–16 years after index pregnancy in women with a history of severe preeclampsia as compared to women with a previous normotensive pregnancy.

## Methods

**Figure 1:**
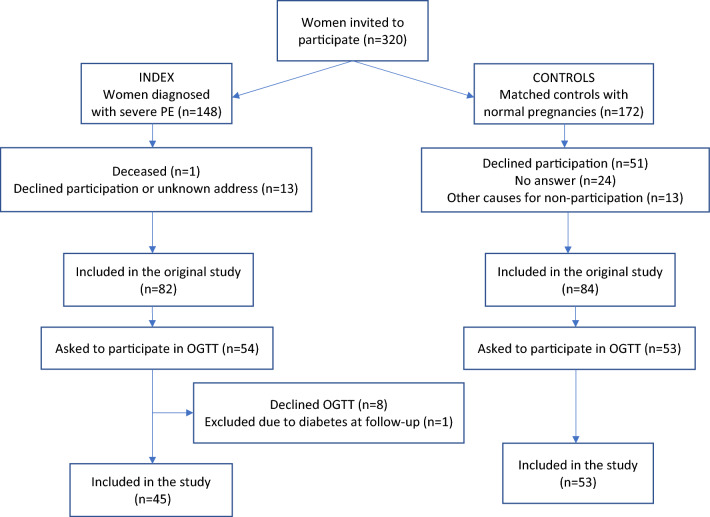
Flow chart specifying inclusion and exclusion criteria of the study population.

The inclusion criteria of this follow-up cohort study were a previous diagnosis of severe PE and having been admitted to Danderyd University Hospital (Stockholm, Sweden) between 1999 and 2004. Participants were identified through the ICD-10 codes O14.1 (severe PE), O14.1A (severe PE with no organ involvement), O.14.1B (severe PE with organ involvement), and O14.1X (severe PE, unspecified) in the patient record system Obstetrix^17^. All women meeting inclusion criteria received an invitation by letter between 2013 and 2016 and were subsequently called up to five times to confirm participation. An inability to establish telephone contact and/or a diagnosis of diabetes mellitus type 1, 2 or gestational diabetes (GDM) during index pregnancy were the only exclusion criteria used. Out of 148 women meeting inclusion criteria, 82 were enrolled in the original study regarding cardiovascular health after PE. From October 2013 onwards, OGTT, serum lipid levels and markers for insulin resistance were added to study the development of prediabetes after PE. In total, 54 cases were invited to do an OGTT, of whom 46 accepted as well as 53 controls matched for age, year of delivery and parity. Control women were selected using medical records, a midwife called the next coming woman who had delivered a child after a normal singleton full term pregnancy, spontaneous onset of labor and cephalic presentation after the index woman delivery. The control women women asked to participate in the study as controls. If a woman declined participation the next woman matching all the inclusion criterions as a control was approached. One index woman had developed type 2 diabetes and was excluded from the study. See Fig. 1.

Data concerning the cases’ and controls´ index pregnancies were retrieved retrospectively from medical records stored in the Stockholm County Council Archive. Diagnoses of PE were verified by an obstetrician (Ellika Andolf) by chart review, and it was ascertained that the pregnancies of the controls had been uncomplicated. A diagnosis of PE was based on the following diagnostic criteria; hypertension (≥ 140/90 mm Hg) after 20 weeks’ gestation accompanied by proteinuria, other maternal organ dysfunction (kidney, liver, neurological complications, or hematological complications) or uteroplacental dysfunction such as intrauterine growth restriction or stillbirth^18^. None of the participants had GDM during the index pregnancy. At the follow-up, 11–16 years after index pregnancy, participants answered questionnaires regarding general health, heredity, lifestyle, and reproduction. Subjects were asked regarding family history (FH) of diabetes, hypertension, stroke, heart disease, myocardial infarction, and cerebral bleeding. Positive FH for these conditions was defined as having a close male relative becoming ill before the age of 55 and/or a close female relative falling sick before the age of 65.

Controls were asked if they ever had PE, one woman had PE after index pregnancy and was therefore excluded.

All participants underwent the same clinical examinations by two specially trained research nurses at follow-up. Waist circumference, height, and weight were measured according to a standardized protocol and BMI was calculated as kg/height^2^. Blood pressure was measured in the right arm in a sitting position using an automatic monitor, after subjects resting for 15 min. Hypertension was defined as either blood pressure ≥ 140/90^19^ or ongoing medical treatment for hypertension. If the blood pressure measured at the follow-up was ≥ 140/90, the measurement was repeated after at least four hours or at a new appointment another day.

The primary outcome measure was the prevalence of prediabetes. We used the definition for prediabetes established by the American Diabetes association (ADA); a fasting blood glucose of 5.6–6.9 mmol/L (100–125 mg/dl), or a 2-h post load plasma glucose of 7.8–11.0 mmol/L (140–199 mg/dl) after drinking 75 g glucose dissolved in water (OGTT) or a HbA1c of 39–47 mmol/mol (5.7–6.4%)^20^. Participants left several venous blood tests including HDL, triglycerides (TG), cystatin C, high-sensitivity CRP (hsCRP), HbA1c, insulin, and C-peptide. Insulin sensitivity was quantified by HOMA-IR and glucose tolerance by FPG and OGTT where plasma glucose was then measured consecutively at 30 and 120 min. HOMA-IR was defined as insulin (mU/L) multiplied by glucose (mmol/L) divided by 22.5.

Plasma glucose and plasma cystatin C were analyzed with DXC800 and LX20 from Beckman Coulter (reagents GLUCm, art no 472500 and Gentian Cystatin C UDR-kit, art no A52761). HsCRP was measured by immunoturbidimetric analysis on Roche Cobas 8000 module c502. HbA1c was measured on Variant II Turbo from Biorad, the instructions from the manufacturer were followed. Insulin and C-peptide were analyzed using Roche Modular E or Roche Cobas e602. HDL and TG were analyzed with LX20 and DXc 800 by Beckman-Coulter. In 2015 the equipment used for analyzing HDL and TG was exchanged to Cobas 8000, Roche.

The metabolic syndrome was defined according to the International Diabetes Federation^21^ as a waist circumference > 80 cm and at least two of the following criteria: triglycerides ≥ 1.7 mmol/l, HDL < 1.3 mmol/l, FPG > 5.6 mmol/L or IGT, blood pressure > 130/85 mm Hg or antihypertensive medication. The prevalence of the metabolic syndrome was compared among index and control women.

The study was approved by the ethical review board; reference numbers 2013/947:31/4, 2015/1085-32.

### Statistical analysis

Data are presented as median and minimum/maximum values for continuous variables while categorical data are presented as numbers (n) and percent (%). Due to a small study population the Mann–Whitney U-test was used to compare index and control women on continuous variables while Pearson’s chi-square was used on categorical data. However, multivariable analyses included single and multiple logistic regression models. In these models the presence of prediabetes and fasting plasma glucose ≥ 5.6 mmol/L were defined as the dependent variables (each dependent was modelled separately) and independent variables were group (i.e., having had PE during pregnancy), family history of diabetes mellitus or CVD, hypertension at follow-up and waist circumference at follow-up. Adjustments were made for waist circumference rather than BMI as it is considered superior in measurement of body fat. One value for TG was considered erroneous (TG = 86.0 mmol/L) and was therefore regarded as a missing value. All data were analyzed using IBM SPSS, version 26 (IBM Inc., Armonk, NY, USA). Statistical significance was defined as *p* < 0.05 (two-sided).

### Ethics approval and consent to participate

The study was approved by the Regional Ethics Review Board, Karolinska Institutet, Stockholm, Sweden; reference numbers 2013/947:31/4, 2015/1085–32. Participation in the study was free of will and all participants provided informed consent to participate in the study. All methods were carried out in accordance with guidelines and regulations.

## Results

Maternal age and parity did not differ between index women and control women at baseline. However, the index women had significantly higher blood pressure (*p* < 0.001) and BMI (*p* < 0.001) at the first trimester of the index pregnancy compared to control women (Table 1). Four women were using tobacco at baseline, two in each group (data not shown).

**Table 1:** Demographic and anthropometric characteristics of study population.

Characteristic	Index women (history of PE, n = 45)	Control women (without PE, n = 53)	*p* value
Baseline			
Age at baseline pregnancy (median, min–max)	33.00, 22.00–42.00	34.00, 22.00–42.00	0.932
Parity			0.919
Primiparous (n (%))	31 (69.9)	36 (67.9)	
Multiparous (n (%))	14 (31.1)	17 (32.1)	
BMI at baseline pregnancy (kg/m^2^) (median, min–max)	23.40, 19.50–39.80	22.20, 17.70–29.80	< 0.001
SBP at baseline pregnancy (mmHg) (median, min–max)	120, 90.00–140.00	110.00, 90.00–135.00	< 0.001
DBP at baseline pregnancy (mmHg) (median, min–max)	74.00, 56.00–101.00	65.00, 50.00–90.00	< 0.001
Follow-up			
Age at follow-up (years) (median, min–max)	45.00, 43.00–49.00	46.00, 36.00–55.00	0.726
Level of education at follow-up:			0.353
Elementary schooling or upper secondary education (n (%))	10 (22.2)	14 (26.4)	
University education or doctoral studies (n (%))	35 (77.8)	37 (69.8)	
Missing values (n (%))	0 (0)	2 (3.8)	
Income at follow-up, SEK/year			0.170
< 600,000 (n (%))	35 (77.8)	38 (71.7)	
> 600,000 (SEK/year) (n (%))	10 (22.2)	11 (20.8)	
Missing values (n (%))	0 (0.0)	4 (7.5)	
SBP at follow-up (mmHg) (median, min–max)	121.00 102.00–169.00	116.00, 94.00–155.00	0.050
DBP at follow-up (mmHg) (median, min–max)	74.00, 56.00–101.00	72.00, 53.00–99.00	0.067
Hypertension at follow-up (n (%))	15 (33.3)	5 (9.4)	0.003
Heredity for DM and/or CVD at follow-up	17 (38.6)	9 (17.3)	0.020
Missing values (n (%))	1 (2.3)	1 (1.9)	
BMI at follow-up (kg/m^2^) (median, min–max)	25.00, 19.70–41.60	22.50, 18.00–33.00	0.001
Waist circumference at follow-up (cm) (median, min–max)	88.00, 70.00–115.00	83.00, 69.00–119.00	0.024
Waist circumference, WHO limits (n (%)):			0.580
− 80	11 (23.2)	22 (41.5)	
81–88	12 (26.7)	17 (32.1)	
89-	22 (48.9)	14 (26.4)	

*P*E Pre-eclampsia, *SBT* systolic blood pressure, *DBP* diastolic blood pressure, *DM* diabetes mellitus, *CVD* cardiovascular disease, BMI body mass index.

All data from baseline pregnancies was retrieved in the first trimester.

At follow up, the distribution of income and educational level were similar in the two groups. Moreover, at follow-up index women more often had hypertension (33.3% vs 9.4%, *p* = 0.003), FH of diabetes and/or FH of CVD (38.6% vs 17.3%, p = 0.020), a higher BMI (median 25.0 vs 22.5, *p* = 0.001) and a larger waist circumference (median 88.0 cm vs 83.0 cm, *p* = 0.024) compared to control women (Table 1). The index women had increased the median BMI compared to baseline levels, which was not the case for the control women.

**Figure 2:**
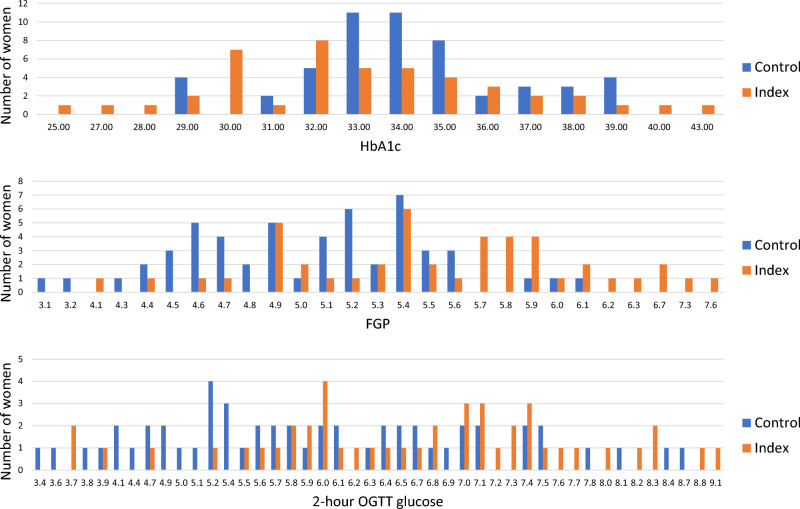
Distribution of HbA1c, FGP and Glucose at 120 min by group.

The prevalence of prediabetes at follow-up was significantly higher in the index group (n = 24, 53.3% vs n = 10, 18.9% *p* = 0.001) compared to the control group. Also, median levels of FPG (median 5.50 vs 5.10, *p* < 0.001) and 2-h OGTT glucose levels (median 6.80 vs 5.80, *p* = 0.005) were significantly higher in the index group compared to the control group. The metabolic syndrome was significantly more common in index women compared to control women (n = 12, 26.7% vs. n = 3, 5.7%, *p* = 0.005). Women in the index group had significantly lower levels of HDL (median 1.6 vs 1.8, *p* = 0.014). However, no significant differences in the median levels of HOMA-IR, triglycerides, C-peptide, insulin, HbA1c or hsCRP were found (Table 2). In Fig. 2 the distribution of HbA1c, FPG, and. 2-h OGTT among control and index women are displayed. Index women had a wider range of HbA1c compared to control women. Also, their levels of FPG and 2-h OGTT were more skewed to the right, indicating higher levels, compared to controls.

**Table 2:** Metabolic characteristics and number of subjects meeting diagnostic criteria for prediabetes and the metabolic syndrome at follow-up 11–16 years after index pregnancy.

Laboratory tests and dysglycemic diagnoses at follow-up	Index women Median, min–max	Controls women Median, min–max	*p* value
FPG (mmol/L)	5.50, 4.10–7.60	5.10, 3.10–6.10	< 0.001
30-min OGTT (mmol/L)	8.30, 5.10–12.60	8.50, 4.80–11.00	0.986
2-h OGTT (mmol/L)	6.80, 3.70–9.10	5.80, 3.40–8.70	0.005
HbA1c (mmol/mol)	33.00, 25.00–43.00	34.00, 29.00–39.00	0.070
Serum insulin (mU/L)	6.30, 2.20–16.00	5.60, 2.00–20.00	0.503
Serum C-peptide (nmol/L)	0.61, 0.38–1.50	0.55, 0.27–1.13	0.079
High-sensitivity CRP (mg/L)	0.72, 0.20–66.30	0.46, 0.20–4.80	0.158
Plasma cystatin C (mg/L)	0.70, 0.54–1.00	0.75, 0.49–1.00	0.090
HDL (mmol/L)	1.6, 1.0–2.5	1.8, 1.1–3.7	0.014
Triglycerides (mmol/L)	0.72, 0.25–1.70	0.63, 0.23–2.20	0.091
HOMA-IR	1.62, 0.53–4.73	1.21, 0.28–4.62	0.071

Meeting ADA's criteria for	n (%)	n (%)	
Prediabetes	24 (53.3)	10 (18.9)	0.001
Meeting criteria for			
Metabolic syndrome	12 (26.7)	3 (5.7)	0.005

*FPG* fasting plasma glucose, *OGTT* oral glucose tolerance test, *HbA1c* glycated hemoglobin, *CRP* C-reactive protein, *ADA* the American Diabetes Association, *T2DM* type 2 diabetes mellitus.

Multiple logistic regression models revealed that index women had an increased risk of having an FPG ≥ 5.6 mmol/L compared to women in the control group (aOR = 7.28, 95% CI 2.44–21.76) when adjusting for heredity of diabetes mellitus and/or CVD, waist circumference and hypertension at follow-up (Table 3). Similarly, index women had an increased risk of prediabetes according to any ADA criteria after adjustments for hypertension, heredity of diabetes mellitus and/or CVD and waist circumference at follow-up (aOR = 4.83, 95% CI 1.80–12.97), compared to control women (Table 4). Similar findings, i.e., an increased risk for prediabetes among index women compared to control women, when adjusting for heredity, hypertension, and BMI at follow-up (instead of waist circumference). Also, among women who developed prediabetes, it was found that control women had a lower BMI at baseline compared to index women (*p* = 0.007, data not shown).

**Table 3:** Multivariate logistic regression with fasting plasma glucose ≥ 5.6 mmol/L as the dependent variable and PE, heredity, hypertension and waist circumference at follow-up as independent variable.

	Crude estimates		Adjusted estimates Model excluding waist circumference		Adjusted estimates Model including waist circumference	
	OR (95% CI)	*p* value	OR (95% CI)	*p* value	OR (95% CI)	*p* value
Group						
Women w/o PE	*Reference*		*Reference*		*Reference*	
Women w/PE	7.18 (2.57–20.07)	< 0.001	7.68 (2.60–22.69)	< 0.001	7.28 (2.44–21.76)	0.013
Heredity						
No	*Reference*		*Reference*		*Reference*	
Yes	1.45 (0.55–3.80)	0.451	0.81 (0.27–2.48)	0.812	0.80 (0.25–2.50)	0.879
Waist circumference follow-up	1.04 (1.00–1.09)	0.054			1.03 (0.98–1.08)	0.195
Hypertension at follow-up	1.93 (0.69–5.41)	0.209	1.23 (0.37–4.04)	0.738	1.11 (0.33–3.72)	0.869

*OR* odds ratio, *CI* confidence interval, *PE* pre-eclampsia.

**Table 4:** Multivariate logistic regression with prediabetes according to ADA criteria as the dependent variable and PE, heredity, hypertension and waist circumference at follow-up as independent variable.

	Crude estimates		Adjusted estimates Model excluding waist circumference		Adjusted estimated Model including waist circumference	
	OR (95% CI)	*p* value	OR (95% CI)	*p* value	OR (95% CI)	*p* value
Group						
Women w/o PE	*Reference*		*Reference*		*Reference*	
Women w/PE	5.12 (2.08–12.59)	< 0.001	5.18 (1.98–13.55)	0.001	4.83 (1.80–12.97)	0.002
Heredity						
No	*Reference*		*Reference*		*Reference*	
Yes	1.15 (0.46–2.91)	0.768	0.73 (0.25–2.11)	0.555	0.73 (0.24–2.22)	0.576
Waist circumference follow-up	1.06 (1.01–1.10)	0.011			1.05 (1.00–1.10)	0.040
Hypertension at follow-up	1.74 (0.64–4.72)	0.281	1.24 (0.39–3.93)	0.713	1.05 (0.32–3.43)	0.576

*OR* odds ratio, *CI* confidence interval, *PE* pre-eclampsia.

## Discussion

Women with PE had an almost fivefold increased risk of prediabetes 11–16 years after the index pregnancy. Since they were young, and still premenopausal with endogenous oestrogen production this should provide protection against insulin resistance^22^. Approximately half (53.3%) of the women had developed prediabetes as compared to 18.9% in the control group. Women with PE had a significantly higher FPG and 2-h OGTT. However, no significant difference in HbA1c was found. FPG, OGTT and HbA1c are considered to partially represent different aspects of glucose metabolism. FPG represents endogenous glucose production by hepatic gluconeogenesis. People with isolated impaired fasting glucose (IFG) thus have a high hepatic insulin resistance. People with isolated impaired glucose tolerance (IGT, increased 2-h OGTT) primarily have a high muscle insulin resistance. Both share a beta cell dysfunction as they do not compensate with sufficient insulin secretion. In IFG the early phase of insulin secretion is inadequate. In isolated IGT there is an impaired insulin secretion in both the early and late phase^23^. HbA1c increases when both FPG and non-fasting glucose e.g., 2-h OGTT are elevated. Thus, among the women with PE we see IFG and/or IGT as well as some with increased HbA1c levels. To compensate for the impaired beta cell function in combination with insulin resistance the insulin secretion during the second phase is increased and often delayed and the insulin hepatic extraction is reduced. However, oestrogen increases the insulin sensitivity which decreases the need for insulin^22^. This may explain why the increase in c-peptide (*p* = 0.079) or HOMA-IR (*p* = 0.071) did not reach significance.

Most of the prediabetic cases in our study fulfilled ADA’s FPG criteria for prediabetes while a smaller portion met the HbA1c criteria. HbA1c is affected by both fasting and post-prandial glucose levels and dependent on red cell turn over. HbA1c has been found to have a higher sensitivity of identifying IGT than FPG alone, but FPG has a higher specificity^24^. The risk of progression to type 2 diabetes is higher in those with both IGT and IFG than HbA1c, IFG or IGT alone^25^. The odds ratio of IFG after PE was 7.28 after adjustment for heredity of CVD or diabetes mellitus, waist circumference and hypertension at follow-up. The odds ratio of prediabetes according to any ADA criteria was lower, 4.83. Thus, such a large proportion of prediabetic women with a history of PE with IFG may reflect a severe metabolic profile with primarily hepatic insulin resistance in these women.

There are some limitations to the study. Several parameters were gathered from questionnaires as well as physical assessments and there was a substantial loss to follow up. It is difficult to achieve a high participation rate in questionnaire- and clinical studies and the loss to follow up increases the risk of bias and decreases the generalizability and reliability of our findings. In addition, the small sample size decreased the power and increases the risk of type 2 errors.

Also, the study does not include data on diet, physical activity level and ethnicity which are important risk factors for diabetes mellitus^26^. However, the study provides important information about these women that would not have been able to be gathered using registers.

A major strength of the study is the measurement of anthropometric data by specially trained research nurses and the gathering of blood samples where diagnosis of prediabetes was based on actual blood glucose levels. This makes the data reliable. Furthermore, the diagnosis of severe PE was based on diagnostic criteria according to ICD-10 and reviewed by a skilled obstetrician (Ellika Andolf) making the diagnosis of PE reliable. The medical records of the women were carefully reviewed before inclusion. By reviewing medical records, it was possible to exclude all women with diabetes mellitus or GDM to eliminate the risk of confounding bias. Another strength is the length of the follow-up. By including only women with severe PE who were treated at the same hospital we decreased the heterogeneity and we had well matched controls.

The findings are in line with the previous studies. In Canada it was found that women with PE had an increased risk of developing prediabetes four years after parturition. They found mean FPG, mean random plasma glucose and mean HbA1c were all significantly higher four years after delivery in women with a history of PE compared to normotensive women. The odds of prediabetes were adjusted for maternal age at delivery, parity, and an index to present socioeconomic disparities. However, the study’s data sets did not include information on family history of diabetes, CVD, or measures of obesity, and they were not able to account for these risk factors in the analytical models^27^. Furthermore, their data were based on indicated glucose testing in 60% of normotensive pregnancies and 67.8% of women with PE. Therefore, they may have missed prediabetes in untested participants.

A prospective cohort study in the Netherlands found that women with self-reported previous hypertension during pregnancy had a significantly higher FPG after the age of 45 than women with a previous normotensive pregnancy. Women with previous hypertension during pregnancy also had a higher prevalence of diabetes after the age of 50. The study was limited by the lack of a clear definition of hypertension during pregnancy since the participants self-reported the prevalence and no differentiation was made regarding PE or gestational hypertension which may have diluted the results^27^.

There were no significant differences in hsCRP, insulin, C-peptide, triglycerides, or Cystatin C. HsCRP and Cystatin C have been associated with prediabetes^28–30^ and risk of developing type 2 diabetes. Insulin and C-peptide are markers of insulin resistance. Therefore, the lack of differences between the groups were surprising, however it may be due to a lack of power to detect such differences because of a small study population. However, women in the index group had a significantly lower HDL which is associated with both IFG and IGT^28^.

Women in the index group had higher BMI in the first trimester. Unfortunately, there was a significant lack of data regarding weight gain during pregnancy why we were not able to analyse this in any meaningful way. Index women also had a larger waist circumference, a higher BMI, and more often hypertension at follow-up. The metabolic syndrome was more common among index women which is in line with previous studies^31^. A high BMI and especially waist circumference are associated with insulin resistance. Waist circumference was significantly associated with prediabetes and adjustment for waist circumference decreased the odds of prediabetes in index women. However, the odds remained high, independent of waist circumference. Insulin resistance is believed to contribute to the pathogenesis of PE irrespective of obesity. Women with PE have higher insulin resistance both during and after pregnancy compared to women with normal pregnancies independent of BMI^5,6^. Subsequently our results may support the hypothesis that women with PE have a adverse metabolic phenotype independent of obesity.

First-degree relatives of patients with type 2 diabetes have an increased risk to develop the disease^32^ and women with a history of PE in our study more often reported FH of diabetes mellitus or CVD. However, in the analysis, heredity did not have a significant impact on the risk of prediabetes. Nor did hypertension have a significant impact on the risk of prediabetes.

The prediabetic period represents an opportunity to intervene to prevent or delay progression to diabetes and adverse events. Annually about 5–10% of people with prediabetes develop diabetes^25^. Lifestyle interventions or metformin therapy lowers the risk of progression to diabetes according to a meta-analysis, by 36% and 26% respectively^24^.

There is a lack of uniform guidelines on how to follow women after PE. There is increased attention regarding CVD risk and follow-up after PE. In Canada special maternity clinics with an interdisciplinary follow-up have been developed. Their emphasis is put on increased information regarding CVD risks as well as support in lifestyle changes^33^. Our findings support the need for a structured follow-up where women need individualised information not only about their increased risk of CVD but also of diabetes. Currently a randomized controlled trial in Australia evaluates three different kinds of follow-up after PE to evaluate the best way to inform women and support their lifestyle changes^34^. According to a study from Canada 123 women with PE had to be followed for five years to detect one case of type 2 diabetes. Numbers needed to screen (NNS) decreased with increased length of follow-up^10^. Indeed, according to a prospective cohort in the Netherlands based on self-reported prevalence of hypertension during pregnancy the NNS to detect one case of type 2 diabetes after the age of 50 was ≤ 22 for women with a previous hypertensive disorder of pregnancy compared to ≤ 45 in women with a normotensive pregnancy^27^.

Population-based screening for type 2 diabetes has not always been associated with reduction in all-cause, CVD or diabetes related mortality^35,36^. However, studies in Denmark^37^ and Sweden^38^ have found significantly decreased mortality and earlier diagnosis of diabetes in middle aged people screened for diabetes when screening is combined with treatment or lifestyle advise. ADA recommends risk-based screening^20^. Therefore, clinicians should consider risk-based targeted screening in all women with a history of PE. As prediabetes is primarily diagnosed in the primary health care system it is important that general practitioners are aware of this increased risk in women with previous PE. Most importantly women with PE should be informed of their increased risk of dysglycemia and receive adequate information and education regarding potential preventive risk-management measures. A healthy lifestyle and a healthy weight reduce the risk of type 2 diabetes and CVD and should be encouraged. ADA recommends considering metformin therapy to selected prediabetes patients; particularly those with a BMI ≥ 35 kg/m^2^, those aged < 60 years, and women with prior gestational diabetes mellitus^39^. Thus, preventative metformin therapy should be considered in a large proportion of prediabetic women with a history of PE.

In conclusion, PE is associated with increased risk of developing prediabetes, independently of heredity, hypertension, and waist circumference at follow-up. PE may allow early identification of women at risk of metabolic disease and present an opportunity to intervene and potentially prevent or delay progression and adverse events. Clinicians should be alerted to the need for preventative counselling and risk-based targeted screening in this group of women.

## Data Availability

The data that support the findings of this study are available, but restrictions apply to the availability of these data. The data were used under approval from the Regional Ethical Review Board which only approved public sharing and presentation of data on a group level, can thus not be made publicly available. Data are however available from the authors upon reasonable request and with permission of the Ethical Review Board. To request data contact Dr. Ellika Andolf at ellika.andolf@ ki.se.
